# Beef Quality Assurance national rancher survey: program participation, best management practices, and motivations for joining future sustainability programs

**DOI:** 10.1093/tas/txac094

**Published:** 2022-08-01

**Authors:** S C Klopatek, A M Cantwell, L Roche, K Stackhouse-Lawson, J W Oltjen

**Affiliations:** Department of Animal Science, University of California, Davis, CA 95616, USA; Institute for Research in the Social Sciences, Colorado State University, Fort Collins, CO 80523, USA; Plant Sciences University of California, Davis, CA 95616, USA; AgNext, Colorado State University, Fort Collins, CO 80523, USA; Department of Animal Science, University of California, Davis, CA 95616, USA

**Keywords:** Beef Quality Assurance, injection site lesions, ranchers, sustainability, survey

## Abstract

Till date, with over 137,000 certified members, the most successful rancher educational program has been the Beef Quality Assurance (BQA) program. The BQA program was established in the mid-1990’s to improve animal health and welfare with a primary objective to reduce the incidence of injection site lesions by instructing producers to administer injections in the neck only. The present study investigated the drivers of this success to inform future rancher education programs around agricultural sustainability. An online multistate survey was administered to cattle ranchers in collaboration with state cattlemen’s associations to better understand rancher motivations for adopting new practices and to gain insight on current involvement in BQA. In total, the survey consisted of 45 questions and was divided into 3 sections: (1) rancher demographics, (2) BQA participation and current best management practice (BMP) application, and (3) willingness to join new rancher educational programs. Data from 842 respondents are including in this study. Of the survey participants, 70% were currently BQA certified or had been BQA certified at one time, and 30% had never been certified. Ranchers who were BQA certified at any time were less likely to administer injections in areas other than the neck compared to ranchers who were not certified (*P <* 0.05), demonstrating the effectiveness of the BQA program. More than 80% of survey respondents who joined the BQA program stated they believed the BQA program improved animal health and welfare on their operation (*n* = 617). Among those who had not joined the BQA program, 40% believed BQA practices did not align with their ranching operation, while 38% had not heard of the BQA program (*n* = 256). The survey indicated that male ranchers, those with more years ranching, those with a larger percent of income coming from ranching, and ranches with larger total acres grazed were more likely to be BQA certified at any time (*P <* 0.05). Finally, ranchers who were BQA certified at any time were more likely to state that joining a rancher sustainability program would be beneficial to their operation. In conclusion, not only did the survey provide valuable insight into BQA program adoption but highlighted how BQA pedagogy and program structure may be a suitable framework for creating future rancher sustainability programs.

## INTRODUCTION

**Figure 1.**
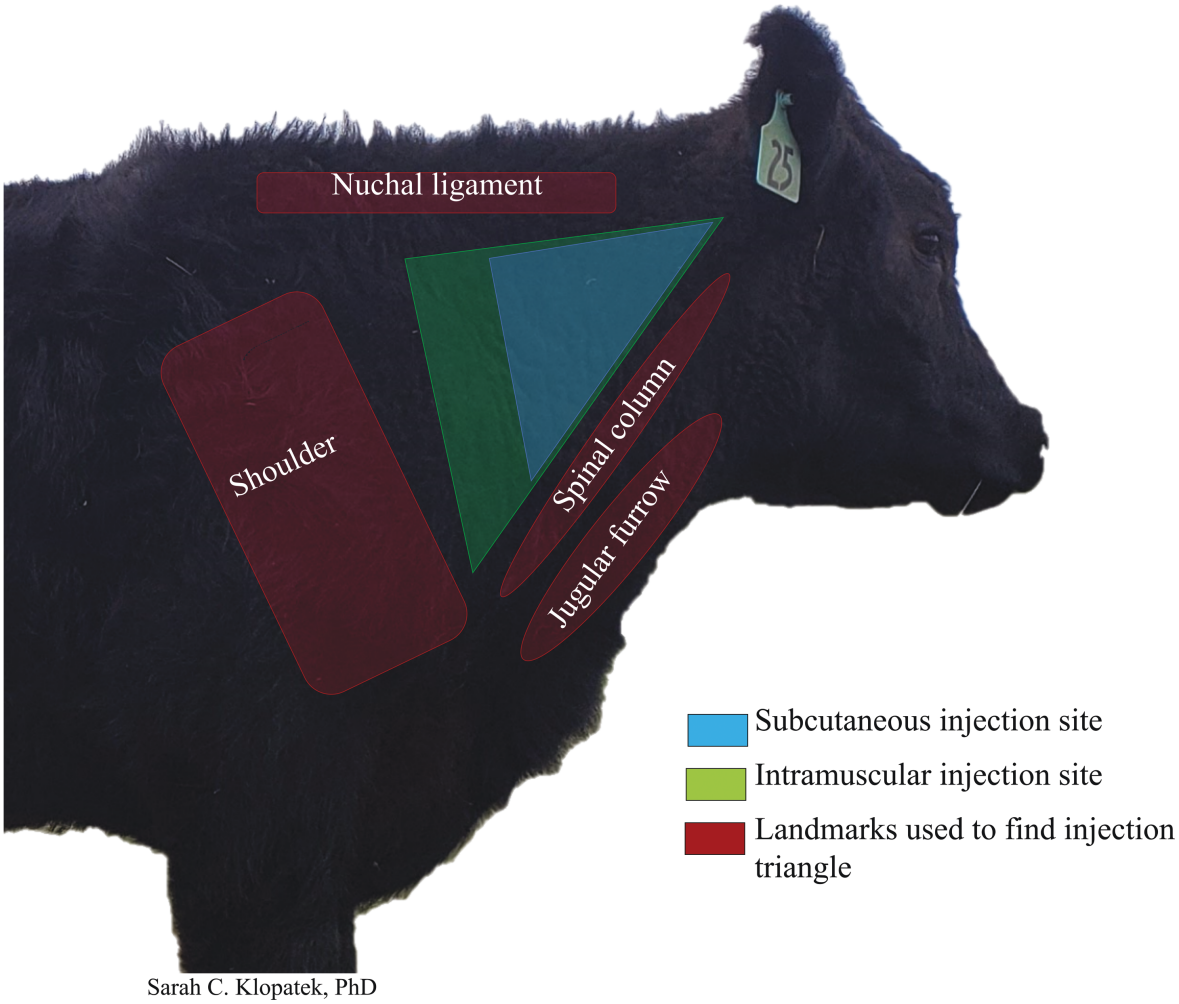
Beef Quality Assurance recommendation to give all injections into the neck only, shown in the green and blue triangle.

To stay competitive in the sustainable foods movement, the beef industry has sought to improve sustainability within their own supply chains. However, before system sustainability can be achieved, sustainability practices must be adopted and implemented at the foundation of the system. With over 725,000 cow-calf producers in the United States (USDA-NASS, 2021), ranchers are the foundation of the beef supply chain. Identifying reasons why a rancher would adopt an educational practice or program is critical for developing sustainability and best management practices (BMP). Although several studies have examined cow-calf producers’ motivations for volunteering for rancher education and conservation programs (Kachergis et al. 2013; Lubell et al., 2013; Roche et al., 2015), no study has investigated the reasons why ranchers choose to be part of the successful rancher educational program, Beef Quality Assurance (BQA). The BQA program was founded in the mid-1990s to improve animal health and welfare, and thereby the beef product itself. A primary objective of the program was to decrease the incidence of injection site lesions found in carcasses, that had a significant impact on the industry costing on average $13.02 (adjusted for inflation at 86.2%; NBQA, 1995) per head. To reduce the incidence of injection site lesions and improve both animal welfare and carcass quality, the program promoted the BMP of administering injections in the neck only (Fig. 1). Since the program’s inception, the prevalence of injection site lesions has decreased by more than 90% (NBQA, 2016). Furthermore, with more than 137,000 individuals currently enrolled in BQA (personal communication, BQA representative), BQA is one of the largest volunteer ranching educational programs to date.

With a high degree of rancher involvement and BMP implementation, the BQA program serves as a model and case study for the development of future rancher educational and sustainability programs. However, the reasons ranchers volunteered to join this program have not been extensively investigated. In addition, other than the National Beef Quality Audit (NBQA, 2016), there is little information available regarding the application of BQA BMPs. To address these knowledge gaps, we implemented a multistate survey investigating BQA certification to improve both the development and adoptability of future rancher education and sustainability programs. Specific survey objectives were: (1) identify reasons producers chose to participate in the BQA program, (2) determine what demographic factors influenced BQA participation, (3) identify factors that influenced adoption of the best management practice of neck-only injections, and (4) determine if there were relationships between BQA certified producers and involvement in a nonmandatory sustainability program.

## Materials and Methods

### Survey Design and Recruitment Procedures

**Figure 2.**
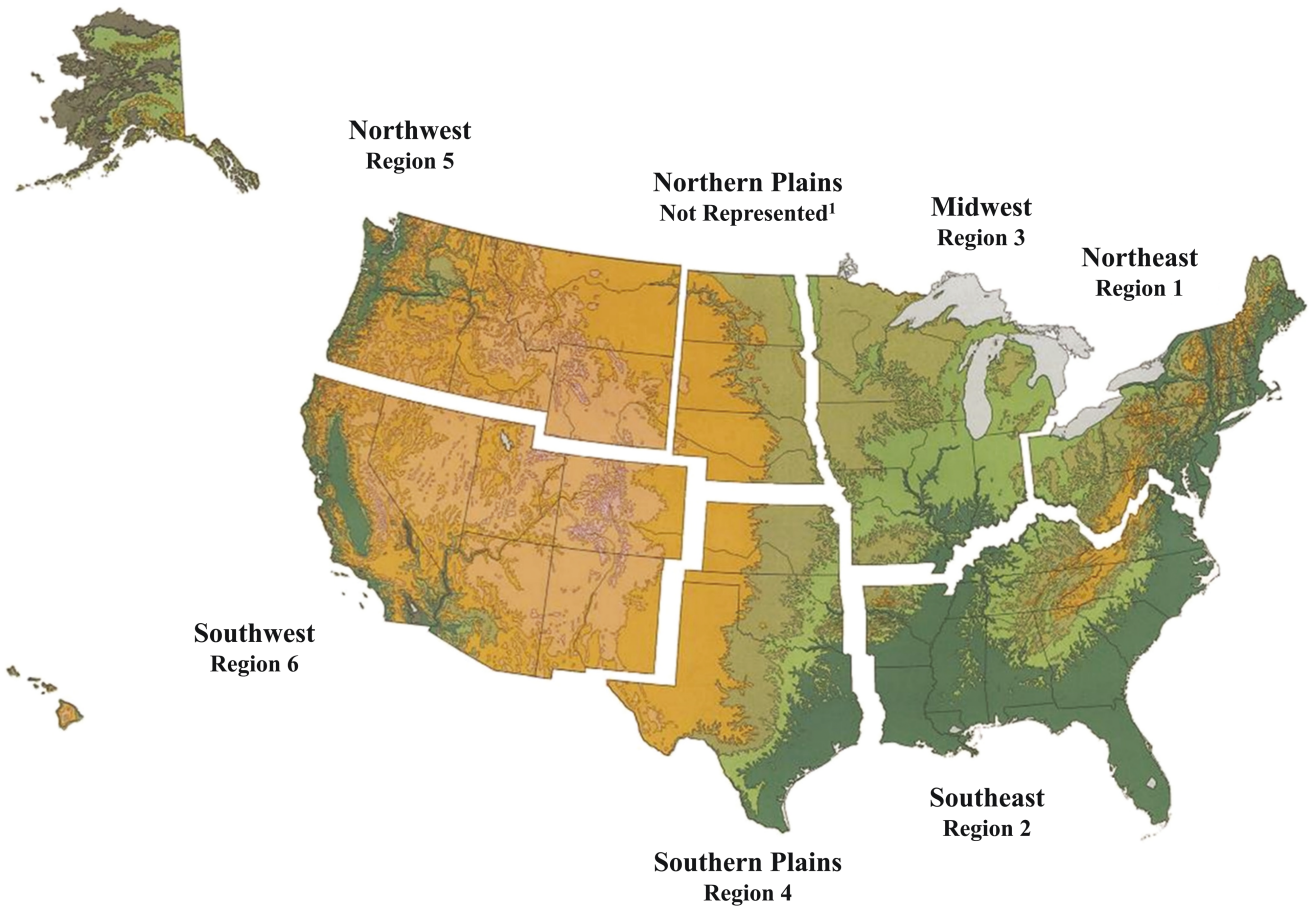
The “Rancher Management Practices” survey was administered to ranchers across six of the seven National Cattlemen’s Beef Association Rancher regions in the United States. The survey was administered from June 1^st^ to December 31^st^, 2019. ^1^Image provided by the National Beef Cattlemen’s Association 2 Ranchers represented in Northwest Region (Region 1), Northern Plains, Midwest Region, Northeast Region, Southwest Region (Region 6: 288), Southeast Region, and southern Plains.

An online survey for ranchers was developed and administered using the platform Qualtrics (Provo, UT). In total, the survey consisted of 45 questions divided into three sections including: (1) Rancher Demographics, (2) Beef Quality Assurance Participation and current BQA practice application, and (3) Willingness to join new rancher educational programs. Survey questions were derived from literature and discussions with collaborating ranchers. After questions were developed, the initial survey was pilot tested with California ranchers located in northern California. Once pilot testing was completed and minor adjustments were made, the final survey was administered online to ranchers across six of the seven National Cattlemen’s Beef Association regions including the Northwest, Southwest, Southeast Midwest, Northern Plains, and Southern Plains (Fig. 2). To recruit ranchers from these regions ranchers were contacted through state cattlemen association listservs. The state cattlemen associations were nonprofit trade organizations serving cattle ranchers, beef producers, and private owners of cattle-grazed properties. Specifically, cattlemen’s associations serve as an in-person and online resource where ranchers receive information and provide feedback on ranching practices and policies. Ranchers on the respective listservs were emailed once a month over a 6-month period with an invitation to complete the survey. The survey was available from June 1st to December 31st, 2019. Ranchers were included in the study if they (1) were a cattle rancher on a state cattleman listserv and (2) currently own cattle. This strategy ensured the survey would capture ranchers with diverse perspectives and management approaches. Although ranchers who did not have access to the internet were excluded, recent studies have determined that internet use is widespread among today’s ranchers (75%–82%; Kachergis et al, 2013; Ghajar et al., 2019). The survey was administered to 1,000 ranchers and 842 answered all questions included in this analysis.

### Operator and Operation Demographics

To provide insights into the key factors shaping BQA membership and BMP practices, descriptive statistics were used to characterize key components adapted from the rangeland decision-making framework from Lubell et al. (2013: Table 1). Table 2 includes the summary information about survey participants including region of operation sex, age, first or multigenerational rancher, and years ranching since reaching age 18. Operation characteristics included percent of income from ranching, existence of succession plan, number of head of cattle, type of land utilized, and total acres grazed. In addition, ranchers were asked about common rangeland and ranch management practices, type of operation, types of certifications, program involvement, vegetation management, and landscape enhancements. Quantitative questions were multiple choice and qualitative questions were fill in the blank. See Supplementary Material for list of survey questions.

**Table 1.** Variables hypothesized to influence Beef Quality Assurance (BQA) certification.

Operator characteristics
• Age
• Years ranching (Since 18 years old)
• Gender
• Have a grazing management plan Time Horizon variables
• Succession Plan
• Generation of ranching
Ranching operation characteristics
• Location of ranch
• Income from ranching
• Participation in ranching certification programs
• Participate in a government landowners assistance program
• Type of land managed and size of operation
• Number of cattle
• Additional income sources on the ranch

**Table 2.** Summary Statistics used in multinomial regression analysis (*n* = 842).

Categorical variables	Frequency	Percent
Age		
18–29	81	9.62
30–39	151	17.9
40–49	122	14.5
50–59	187	22.2
60–69	192	22.8
Over 70	109	13.0
Years ranching (Since 18 years old)		
0–5	99	11.8
6–10	106	12.6
11–20	172	20.4
21–30	154	18.3
More than 30 years	311	36.9
Gender		
Male	624	74.1
Female	218	25.9
NCBA^1^ Region		
1-Northeast	361	42.9
2-Southeast	52	6.18
3-Midwest	18	2.14
4-Southern plains	55	6.53
5-Northwest	88	10.5
6-Southwest	251	29.8
Succession Plan		
Yes (Includes “in progress”)	281	33.4
No	561	66.6
Generation of Ranching		
First	275	32.7
Multigenerational	567	67.4
Percentage of income from ranching		
1–25%	445	52.9
26–50%	206	24.5
51–75%	87	10.3
76–100%	104	12.4
Programs: Humanely raised, verified source, NHTC, GAP		
Participating	243	28.9
Not Participating	599	71.1
Programs: Grass-Fed, All Natural, and/or Certified Organic		
Participating	146	17.3
Not Participating	696	82.7
Participate in a government landowners assistance program		
Yes	503	59.7
No	339	40.3
BQA Certified		
Yes	619	70.0
No	265	30.0

National Cattlemen’s Beef Association.

Examples of land management programs included USDA or Natural Resource Conservation Service (NRCS), such as the Environmental Quality Incentives Program (EQIP), Conservation Stewardship Program (CSP)].

According to the BQA program guidelines, to be considered current in BQA certification participants needed to have completed the certification process within the last 3 years regardless of the previous certification. To become BQA certified producers must take an online or in-person course taught by a state BQA coordinator and/ or trainer. Among the survey participants, 70% were certified or had been BQA certified at one time (*n* = 589), and 30% had never been certified (*n* = 253). Specifically, 24% of survey participants were currently certified and had enrolled in the BQA program within the last 3 years, 35% were previously certified and had recertified in the last 3 years, 24% had not participated in BQA, 11% had been certified at one time but never recertified (*n* = 100), and 7% had gone to a BQA program but never certified. For analysis, BQA participants were grouped into BQA certified (including certified at one time) and those who never certified and/or never participated.

### Current BQA Best Management Practices

Participants who were currently BQA certified or had been certified at one time were asked to select their top three reasons for joining the BQA program. In addition, participants were asked to rank on a 1–5 scale how beneficial the BQA program had been for their ranch. If the survey participant had never been BQA certified they were asked why they chose not to be a part of the program. To identify if ranchers were currently following BQA guidelines survey participants were asked about vaccination administration along with other BQA-specific practices. Specifically, ranchers were asked where they administered antibiotic, vaccine, and hormone injections.

To conclude the survey, ranchers were asked if they were willing to participate in a nonmandatory “beef sustainability” rancher education program that would be beneficial for their operation. Respondents had the answer choices of yes, no, and unsure.

### Statistical Analysis

All analysis was conducted using Stata version 16.1 (StataCorp 2019). The frequency tables for variables in all models are summarized in Table 2. Binomial Logistic regression was used to identify the characteristics that best predict BQA membership (those currently certified or certified at one time compared to those who were never certified). Multinomial logistic regression was used to predict the impact of BQA membership on medical injection decisions (response categories: in the neck only (reference category), other location only, or both neck and other locations) controlling for age, gender, having a succession plan in place, generation of ranching, years of ranching, percent of income from ranching, number of grazed areas, and land assistance program participation. Multinomial logistic regression is best used for dependent variables with nominal outcomes. In multinomial logistic regression, positive coefficients represent an increased probability of choosing one decision (i.e., “other only” and “neck and other” locations) relative to the reference/baseline (i.e., “neck only”). Lastly, binomial logistic regression was used to predict whether BQA membership impacted rancher’s perceived benefits of joining a nonmandatory sustainability program (response categories yes, no or unsure with no and unsure combined for statistical purposes). A *P* < 0.05 statistical significance level was throughout the analysis.

## Results

### Demographics

For demographics regarding participants’ age, sex, location, and ranching operation characteristics, see Table 2.

### BQA Participation

**Figure 3.**
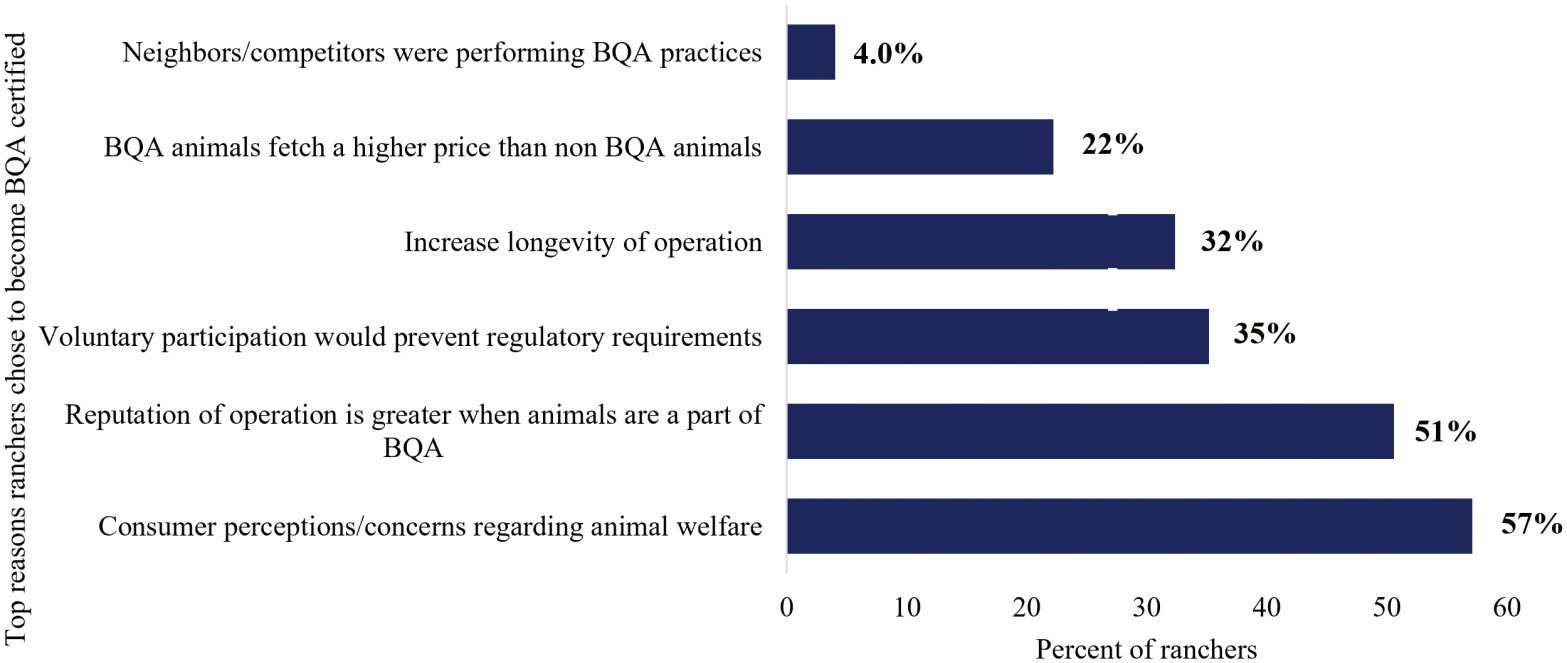
Top three reasons ranchers chose to become Beef Quality Assurance (BQA) certified (*n* = 589).

**Figure 4.**
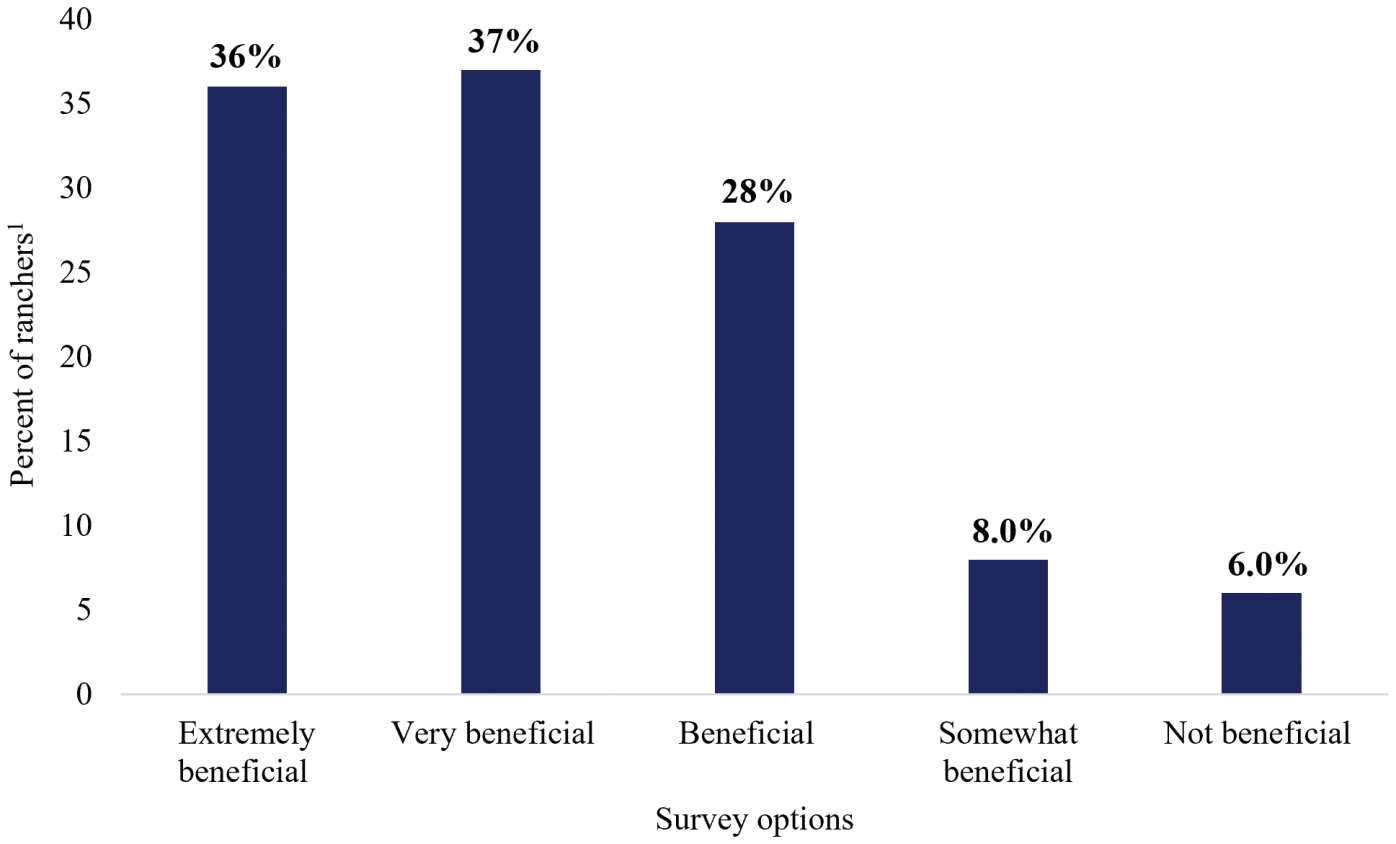
Survey responses to how beneficial becoming Beef Quality Assurance (BQA) certified was to individual operation (*n* = 560).

We first sought to identify rancher motivations for becoming BQA certified. For those who had participated in the BQA program (*n* = 589), the most cited motivations for joining BQA were; (1) to improve animals’ health and welfare, (2) consumer perceptions/demands/concerns about animal welfare, and (3) reputation of their operation is greater when animals are a part of BQA (*n* = 589; Fig. 3). Other reasons ranchers participated in BQA included a belief that voluntary participation would prevent regulatory requirements, BQA would increase the longevity of their operation, and BQA animals would fetch a higher price. In addition, 4% of ranchers participated in the program because neighbors/competitors were performing BQA practices (*n* = 617). When BQA participants were asked if the BQA program had been beneficial to their ranching operation, 26% stated joining the BQA program had been extremely beneficial to their ranching operation, 32% stated very beneficial, 29% beneficial, 10% somewhat beneficial, and 3% stated BQA had not been beneficial (*n* = 617). Of the BQA participants surveyed when asked if there were any sections in the BQA guidelines that were not feasible or helpful only 20 producers listed program concerns. Of the BQA ranchers that had program feasibility issues over 80% of the issues were related to weaning or vaccination procedures. When all surveyed ranchers (BQA and non-BQA certified; *n* = 842) were asked for their top three reasons for adopting a new practice, more than 63% stated profitability, followed by improved animal health (62%), and benefits outweigh effort of practice (41%). These results contrasted with the reasons for joining the BQA program, where profitability ranked 6th as a reason for BQA participation. Of the survey takers that were BQA certified, 73% stated joining the program was extremely or very beneficial, 28 stated beneficial, and only 14% stated somewhat beneficial or not beneficial (Fig. 4, *n* = 560).

**Figure 5.**
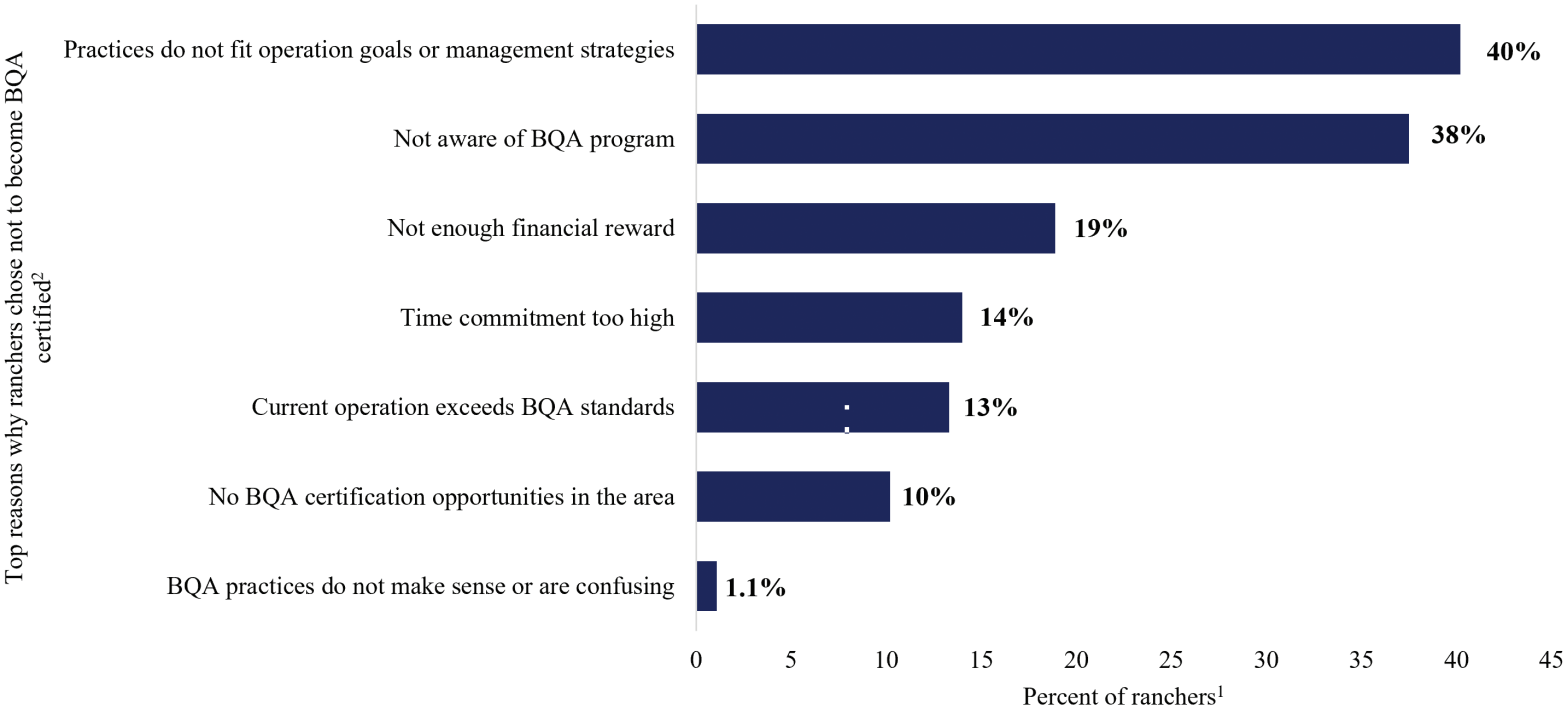
Top three reasons non-BQA certified ranchers chose not to participate in the BQA program (*n* = 253).

For the survey participants who did not join BQA, 40% claimed they did not join the program because the practices did not fit their operation’s goals or management strategies, 38% of participants reported they had not heard of BQA, 19% stated there was not enough financial reward, 13% stated their operation exceeds BQA standards, 14% stated the time commitment was too high, 10% stated there were no BQA certification opportunities in the area, and 1% of participants stated that BQA practices did not make sense or were confusing (*n* = 256; Fig. 5).

### BQA and Best Management Practices

**Figure 6.**
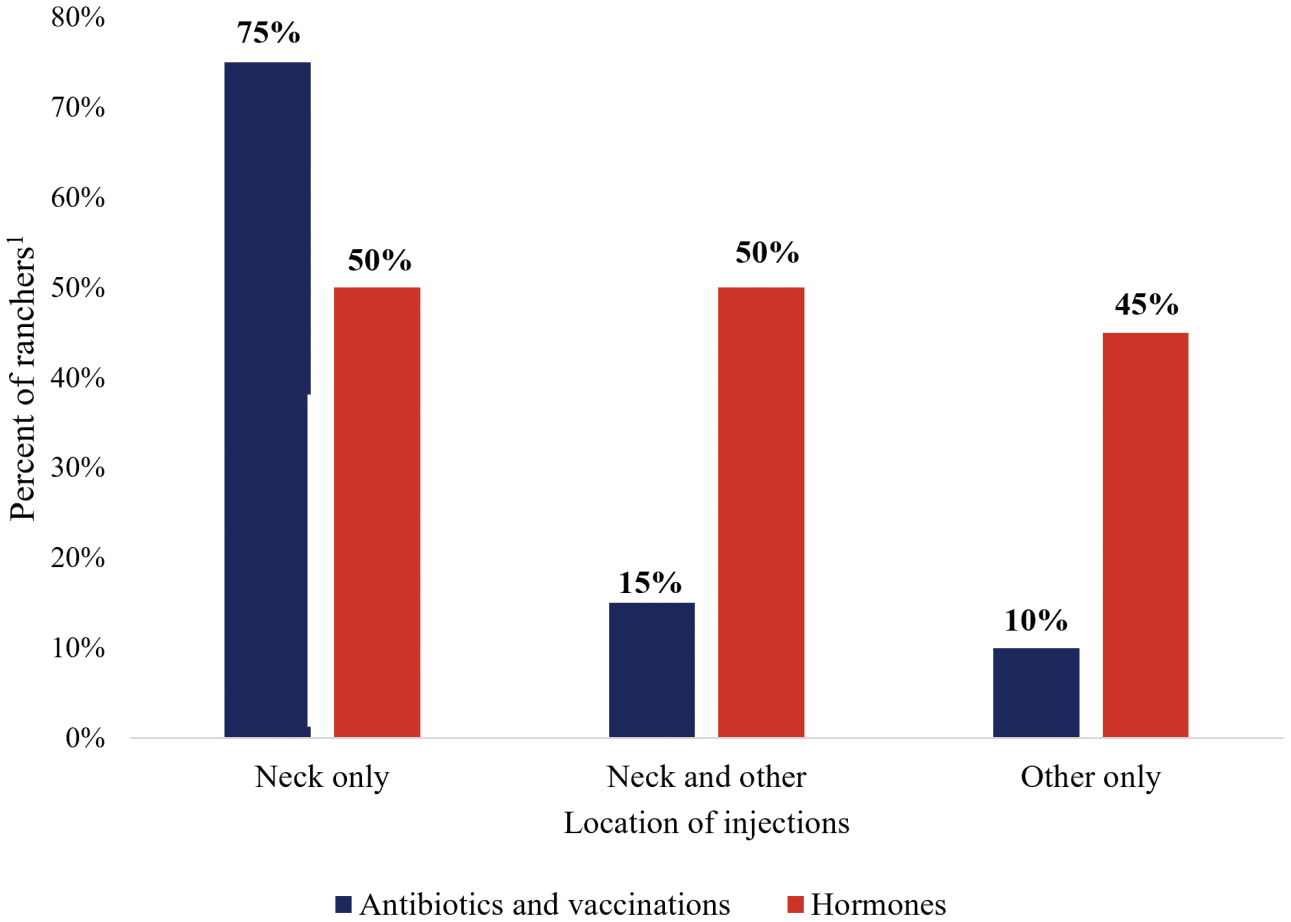
Rancher responses to survey question “Where do you give vaccine/hormone/antibiotic injections for beef cattle?” (Hormone injections n=399 and Antibiotics and Vaccination injections *n* = 783). ^1^Includes BQA and non-BQA member.

The core BMP taught by the BQA program was to administer all injections (antibiotics, vaccinations, and hormones) in the neck only (Fig. 1). The principal reason for injecting in the neck was to reduce the incidence of injection site lesions in valuable retail cuts. In the current survey, of the ranchers (BQA and non-BQA) who administered antibiotics or vaccines 75% administered injections in the neck only (Fig. 6), while 15% injected in the neck but also administered injections in additional areas on the animal (i.e., rump, tail-head, shoulder), and 10% of ranchers administered in areas other than the neck exclusively. For vaccination injections, 51% of ranchers administered the injections in the neck only, and 5% administered injections in the neck and other locations. In contrast to antibiotic and vaccine injections, 44% of ranchers (BQA and non-BQA certified) injected reproductive hormones exclusively in areas other than the neck.

Multinomial logistic regression was used to determine whether there were statistical differences between the chosen site of injections (“neck only”-reference category, “neck and other” location, or “other only”) between BQA certified and non-BQA certified individuals (Table 3). Being BQA certified at any time significantly decreased the probability of ranchers choosing “other only” locations over “neck only” (*P <* 0.05). When examining the location of injections for antibiotics and vaccinations, BQA members were significantly less likely than non-BQA members to choose “neck and other” (*P <* 0.05) and “other only” (*P <* 0.05) locations over “neck only”. The multinomial logistic regression model also identified the percent of ranching income as a factor influencing injection site location choices: those receiving a larger proportion of income from their cattle business were less likely to give antibiotic and vaccination injections in “neck and other” (*P <* 0.05) and “other only” (*P <* 0.05) relative to “neck only”. Additionally, the model identified ranchers with a greater number of cattle and older ranchers less likely to administer antibiotic and vaccination injections in the “other only” relative to “neck only” (*P <* 0.05 and *P <* 0.05, respectively).

**Table 3.** Factors affecting non-recommended injection site locations (“neck and other” and “other only”) relative to the best management practice of “neck only” location (reference category). Effects were analyzed using individual multinomial logistic regressions.

	Injection Type^1^					
	Hormones			Antibiotics and vaccinations		
Factors	Coefficient	Std. err.	*P*-Value	Coefficient	Std. err.	*P*-Value
Neck only	(reference category)					
Neck and other						
BQA certified^2^	−0.22	0.68	0.74	−1.39	0.24	<0.01
Age	−0.17	0.25	0.48	−0.34	0.11	<0.01
Male	0.23	0.64	0.37	0.14	0.26	0.59
Succession plan	−0.38	0.51	0.46	0.40	0.24	0.11
Multigenerational	0.47	0.66	0.48	0.31	0.27	0.94
Number of cattle	<0.01	<0.01	0.46	-<0.01	<0.01	0.61
Acres grazed	<0.01	<0.01	0.47	-<0.01	-<0.01	0.84
Land assistance	−0.39	0.54	0.47	−0.12	0.23	0.59
Percent income from Cattle	0.65	0.25	0.01	−0.07	0.12	0.55
Other Only						
BQA certified^2^	−0.62	0.27	0.02	−0.73	0.33	0.03
Age	0.09	0.10	0.34	0.28	0.12	0.02
Male	−0.75	0.27	0.78	0.06	0.36	0.87
Succession plan	0.08	0.23	0.72	0.50	0.32	0.12
Multigenerational	−0.06	0.27	0.82	−0.35	0.33	0.12
Head of cattle	<0.01	<0.01	0.14	<0.01	<0.01	0.01
Acres grazed	<0.01	<0.01	0.94	<0.01	<0.01	0.32
Land assistance	−0.39	0.22	0.16	−0.18	0.30	0.54
Percent income from Cattle	0.10	0.12	0.38	-0.50	0.22	0.03

Survey participants who administered hormone injections n=399. Survey participants who administered antibiotics and vaccinations injections *n* = 783.

BQA certified includes survey takers that are currently certified or have been certified at one time.

In addition to the BMP practice of injecting in the “neck only”, other BQA program BMPs included fence line weaning (separating the cow and calf via a fence), castrating calves prior to three months of age, waiting 45 days after weaning to ship claves, and establishing a heard health plan with a veterinarian (Table 4; *n* = 842). Of the BQA certified ranchers, 57% stated they fence lined weaned their calves compared to 49% for non-BQA certified ranchers (*P <* 0.05). In terms of castration practices, 55% of BQA ranchers stated they castrated their calves prior to 3 months of age compared to 52% of non-BQA members (*P >* 0.05). In total, 51% of BQA members waited 45 days to ship weaned calves compared to only 25% of non-BQA members (*P <* 0.05). When it came to establishing a herd health plan with a veterinarian, 47% of BQA ranchers had established a veterinary plan while 31% of non-BQA certified ranchers had established a plan (*P <* 0.05). In terms of unsanctioned BQA practices, only 4.6% of BQA ranchers and 7.9% of non-BQA ranchers stated they performed darting on their cattle operations (*P <* 0.05).

**Table 4.** Chi-square: Adoption of BQA and non-BQA recommended management practices performed by BQA-certified^1^ and non-BQA-certified ranchers (*n* = 842).

Cattle management practices X BQA status	BQA certified	Percent	Not BQA certified	Percent	Χ^2^
Recommended practice					
Fence line weaned^2^	335	56.9	125	49.4	4.0*
Castrate before 3 months of age	321	54.5	131	51.8	0.5
Waiting 45 days after weening to ship calves	300	50.9	63	24.9	48.9*
Established herd health plan with veterinarian	277	47.0	79	31.2	18.1*
Non-recommended practice					
Shipping calves 1-7 days after weaning	90	15.3	62	24.5	10.2*
Darting for injections	69	11.7	15	5.90	6.6*
Shipping calves 8-45 days after weaning	83	14.1	60	23.7	11.6*
Castrate after 3 months of age	164	27.8	73	28.9	0.1
**Total**	**589**	**100**	**253**	**100**	

Certification includes those currently certified and those certified at any time in the past.

Fence line weaned indicates separating dams and calves via a fence.

*P <* 0.05.

### Factors Affecting BQA Certification

Logistic regression was used to identify which factors increased the likelihood of ranchers joining the BQA program. Factors that increased the likelihood of becoming a BQA member were total grazing area, percent of income from ranching, region, years ranching, and gender (*P <* 0.05; Table 5). As expected, those with larger grazing areas and those who received a greater proportion of their income from ranching were more likely to be BQA certified. In addition, ranchers in Region 1 (Northeast; Fig. 2) were more likely to be BQA certified than the other regions surveyed. Interestingly, men were more likely to be BQA certified than women. Factors that did not affect BQA certification included age (*P =* 0.12), maintaining a succession plan *(P =* 0.34), and number of cattle owned (*P =* 0.42). No statistical relationship was identified between participation in a land assistance program (e.g., USDA Natural Resources Conservation Service (USDA-NRCS) financial and technical assistance programs) and being a BQA member (*P =* 0.78). Although years of ranching increased the likelihood of participating in a BQA program, being either a first or multigenerational rancher had no effect on BQA membership (*P =* 0.34).

**Table 5.** Variables influencing BQA certification^1^ among surveyed ranchers. Effects were analyzed using individual logistic regressions (*n* = 842).

Variable	Odds	Std. Error	P-value
Age	1.17	0.14	0.12
Gender	0.57	0.14	0.03
Years Ranching	1.34	0.15	0.01
Region 1	9.11	2.67	<0.001
Having a succession plan	0.81	0.19	0.34
Multi-generation rancher	1.34	0.15	0.34
Total Grazing Acres	1.34	0.15	0.03
Head of Cattle	1.00	<0.001	0.42
Land Assistance Program	0.65	1.00	0.78
Percent of Income From Cattle	1.33	0.16	0.01

BQA certified includes survey takers that are currently certified or have been certified at one time.

BQA was designed to help ranchers engage in BMPs to improve the sustainability of their operations through ensuring cattle health. To assess if BQA membership influences the likelihood of joining other sustainability programs, we asked participants if participating in a voluntary sustainability program would be a benefit to their operation and regressed their answers on factors, including BQA membership, that might influence that belief (*n* = 705; Table 6). Over 50% of all survey respondents agreed that participating in a voluntary “beef sustainability” rancher education program would be beneficial for their operation, while 30% said they were unsure, and 20% disagreed. For analysis purposes, the answers of no and unsure were combined. Table 6 shows that ranchers who were BQA certified were more likely to agree that participation in a sustainability program would be beneficial to their operation.

**Table 6.** Variables influencing rancher views on potential new voluntary sustainability programs (*n* = 705). Effects were analyzed using individual logistic regressions for the question “Would a voluntary sustainability program benefit your operation” (yes or unsure/no response categories). ^1,2^

Variable	Odds	Std. Error	P-value
BQA Member	0.71	0.34	0.02
Age	−0.26	0.12	0.04
Male	0.16	0.33	0.61
Years Ranching	<0.01	0.15	0.98
Generation Ranching	-0.60	0.35	0.66
Head of Cattle	<0.01	<0.01	0.89
Total Acres Grazed	<0.01	<0.01	0.36
Participate in a Land Assistance Program	0.58	0.29	0.06
Larger Percent of Income from Cattle	0.14	0.14	0.31

Answers included yes and no or unsure (no and unsure were combined for analysis purposes).

In total (BQA and non-BQA certified ranchers) 370 selected yes, 66 selected no, and 259 survey takers selected unsure.

## Discussion

### Factors and Motivations for Joining the BQA Program

The Beef Quality Assurance program has become one of the most successful volunteer rancher education programs (NBQA, 1995, 2016). The program’s success has been dictated not only by the high matriculation of participants (over 137,460 currently registered, personal communication with BQA representative), but also the high adoption rate of the program’s targeted BMP to administer injections in the neck only (NBQA, 2016). Understanding why ranchers chose to certify in BQA is key to the continued success of BQA and aids in the development and implementation of future rancher educational and sustainability programs. In the present study, ranchers stated their principal reason for adopting any new agricultural practice would be for profitability. This result is consistent with several other studies that determined economic factors (Smit and Skinner 2002; Kachergis et al., 2013; Yung et al., 2015) as the key determinant for implementing new agricultural practices. However, unlike other rancher volunteer programs, such as environmental quality incentive programs (EQIP; funded by USDA-NRCS), the BQA program uniquely focuses on animal health. Though profitability was a key motivation for adopting a new agricultural practice, BQA-certified ranchers stated their number one reason for joining BQA was to improve the health and welfare of their animals (Fig. 3). Interestingly, less than 14% of BQA certified ranchers rated profitability as their number one reason for joining BQA. Despite the low financial interests in joining BQA, over 70% of BQA-certified ranchers stated joining the program had been extremely or very beneficial to their ranching operation (Fig. 4). These results demonstrated the uniqueness of BQA’s success and indicated animal health as a critical factor in the rancher decision-making process.

In addition to asking ranchers why they joined a ranching program, multinomial logistic regression was used to understand the characteristics that distinguish BQA members from nonmembers. The model identified ranchers who generated greater levels of income from ranching were more likely to be BQA certified. This is consistent with previous studies that have shown level of income, capital, and access to labor to be positively correlated with the adoption of new ranching practices (Rowan and White, 1994; Kara et al., 2008, Lamba et al., 2009). In terms of diversification of income, a survey in California determined ranchers with higher numbers of off-ranch income sources were more likely to participate in conservation programs (Lubell et al., 2013). The present study did not identify a relationship between diversification of income and BQA participation. The discrepancies between the previous studies and the current study may be due to different motivational factors for joining a conservation program vs. joining a program focused on animal health.

Previous studies have identified the scale of production (i.e., number of head and number of acres operated), as an indicator for a new program or practice adoption (Thurow et al., 2000; Kreuter et al. 2004; Lubell et al., 2013; Roche, 2016). Specifically, larger operations were more likely to try new practices and programs due to greater economically viable and decreased economic risk. The present study was consistent with these findings for ranchers with more grazing acres were more likely to be BQA certified (Table 5). However, the study did not identify a relationship between number of head and BQA membership (*P =* 0.42). Upon further analysis, the number of head was determined to be weakly correlated with grazing acres (*r =* 0.23; not depicted in Tables). This low correlation between land space and head of cattle may be due to differences in regional stocking densities. For example, in the Western U.S., lower quality rangeland results in lower stocking densities compared to higher stocking densities in the Eastern U.S. where ranchers have increased access to more nutrient dense grazing.

Time Horizon variables (i.e., ranching generation and succession planning) have been positively associated with the adoption of conservation programs (Mishra and El-Osta, 2007; Lubell et al., 2013). In the present study, neither generational status nor having a succession plan influenced the likelihood of BQA participation (Table 5). However, those who ranched for longer periods of time were more likely to be BQA certified. One possible explanation for the decreased participation among less experienced ranchers may be due to decreased awareness of the BQA program. Younger ranchers may have decreased exposure to ranching information sources such as other ranchers, technical service provider trainings, or extension and outreach support organizations as compared to more experienced ranchers. This decreased exposure may have resulted in less experienced ranchers having a lack of understanding of how the potential benefits a certification program like BQA could contribute to the success of their operation. To continue the positive trajectory of the BQA program and to be proactive in future ranching education programs, it is recommended that more effort and outreach be put forth to target individuals with less ranching experience and those with limited available resources.

Type of land (i.e., public or private) used for ranching has been identified as an influencing factor for joining conservation programs. Previous studies have indicated that ranchers who owned greater amounts of private land (opposed to public land) were more likely to join conservation and education programs (Peterson and Coppock, 2001; Neill et al., 2007). Furthermore, these studies indicated that ranchers were less likely to put time, money, or energy into land which they do not own (Peterson and Coppock, 2001; Neill et al., 2007). Due to the low variability of public and private land ownership, the current study was unable to indicate a relationship between the type of land grazed and BQA participation. However, unlike conservation programs that dictate land standards and or benchmarks, the BQA program principally requires action to the animal (i.e., administering injections to the neck), not action to the land (i.e., riparian management). Unlike land that could either be publicly leased, privately leased, or privately owned, all cattle were privately owned by the ranchers. Thereby, it was hypothesized that public land ownership would not be a contributing factor to whether a rancher would or would not become BQA certified.

The number of niche market operations in the United States has been increasing rapidly (Nielsen, 2018). Despite the growth in this sector of beef production, limited data have been produced regarding niche market producers’ motivations for program adoption. Of the surveyed ranchers, 18% participated in either “grass-fed, natural, or organic” programs. Interesting, less than 45% of ranchers participating in these niche market programs were BQA certified (*n* = 65). In contrast, producers who reported being in the niche category’s “humanly raised, verified source and age, non-hormone treated cattle or Global Animal Partnership” resulted in a higher BQA certification of 60% (*n* = 165). The majority of cattle in the “humanly raised, verified source and age, nonhormone treated cattle or Global Animal Partnership” programs were most likely sold to feedlots who were either focused on overseas export or domestic niche production. Ranchers in these programs may have found BQA certification to add value in their operation, particularly when selling cattle at auction. However, ranchers in the “organic, grass-fed, and natural” niche programs may have retained ownership of their cattle throughout the animals’ lifecycles and most likely needed to adhere to additional animal welfare standards (e.g., American Grass-fed Association) than producers in other niche programs. Therefore, it was hypothesized that producers in these programs may not see added value from joining additional programs like BQA.

### Best Management Practices in the Beef Quality Assurance Program

When survey participants were asked where they administered antibiotics and vaccinations, 85% of those who were BQA certified administered antibiotics in the neck only compared to 66% of those not BQA certified (Fig. 6). In addition, BQA-certified ranchers were less likely than non-BQA certified ranchers to choose nonrecommended BQA practices over best management practices (Table 4). A possible reason producers (BQA and non-BQA certified) may not have administered shots in the “neck only” may have been due to the lack of infrastructure. For example, smaller scale ranchers may have lacked simple cattle handling equipment such as a headgate, that would have allowed for easy access to the neck. To accommodate these ranchers, the BQA program may need to increase awareness on how ranchers with limited animal handling resources can administer shots in a safe and effective mannerism. Although more outreach and education will be needed to further reduce the incidence of injection site lesions, this survey demonstrates the BQA program is effective at increasing the adoption of targeted BMPs. Furthermore, with 66% of non-certified BQA ranchers administering injections in the “neck only”, it can be suggested that BQA BMP have transferred to noncertified ranchers as well.

In terms of hormone injections, an informational disconnect was observed between BQA teachings and on-ranch practices. In contrast to vaccines and antibiotics, only 56% of BQA certified ranchers and 39% of noncertified BQA ranchers stated they administered hormone injections in the neck only. Although BQA certified ranchers were less likely to administer hormone shots in “other only” relative to “neck only” locations, both BQA certified and noncertified ranchers were likely to administer hormones in “neck and other” relative to “neck only” locations. However, the observed differences in injection practices between hormones and antibiotics/vaccinations may be due to variations in pharmaceutical approvals for specific purposes. Presently, some pharmaceuticals used for breeding purposes are approved for tail-head/rump administration. Therefore, future BQA programs need to address whether animals considered for breeding purposes can receive hormone injections outside the neck area.

Compared to injection site BMPs, other BQA BMPs, such as fence line weaning, castration prior to three months of age, waiting 45 days to ship calves post weaning, and development of a heard health plan with a veterinarian, were implemented at lower rates among all survey takers (Table 4). However, a higher percentage of BQA-certified ranchers participated in these BQA-sanctioned practices as compared to non-BQA ranchers. The lower participation rate for these BMPs may be due to feasibility or lack of knowledge. For example, some ranching operations may be nutritionally or land restricted resulting in the inability to wait 45 days before shipping weaned calves. This can be the case when forage nutrition declines and the ranch can no longer support the nutritional needs of both the cow and her calf without the aid of hay or grain supplementation. To avoid the additional costs of supplementation ranchers may load calves onto trucks the day of or soon after weaning. Although, the BQA program has been successful in reducing the indigence of injection site lesions, more emphasis and outreach may be needed to increase the adoption rate of other BQA-sanctioned BMPs.

### Relationship to a Sustainability Program

With 80% of ranchers having a positive or neutral view of beef sustainability program participation, there appears to be a willingness for ranchers to engage in future sustainability programs (Table 6). This insight is consistent with the overall growth within the beef sustainability sector. For example, from 2018 to 2021, the United States Roundtable for Sustainable beef saw a 54% increase in income from memberships and sponsorships. In the present survey, ranchers stated they became BQA certified because they believed that the program was beneficial for the health and welfare of their animals, and thereby good for their business. This ethos may translate to motivations for joining sustainability programs. Ranchers may have observed the positive effects of implementing BQA BMPs on their ranching operations and, that may have resulted in their more positive association of sustainability programs compared to those that were not BQA certified. Therefore, the BQA modus operandi may be a suitable structure for future sustainability programs.

## Conclusion

The present survey was the first of its kind to directly ask ranchers across the U.S. about the adoption and efficacy of the Beef Quality Assurance certification program. First, the study found that although there were similar factors driving ranchers to join either the BQA program (become BQA certified) or the conservation program, the principal rancher BQA adoption driver was animal health and welfare. Second, the survey determined that BQA-certified ranchers were more likely to agree that joining a volunteer sustainability program would be beneficial to their operation than non-certified ranchers. The relationship between BQA and sustainability participation demonstrated how aspects of the BQA program may serve as a model for an industry wide sustainability program. Finally, although the BQA program has been highly successful at reducing injection site lesions the survey revealed information gaps and potential areas for improvement. Other BQA BMP including 45-day weaning, castrating prior to 3 months, and establishing a herd health program with a veterinarian were adopted at a much lower rates than the principal BMP of injecting in the neck only. In addition, factors that decreased the likelihood of becoming BQA certified were gender, years ranchers (newer ranchers), and ranchers who received less income from ranching. Therefore, additional resources and outreach efforts should be extended to ranchers in these demographics to increase BQA and BMP adoption. Overall, to further the understanding of ranching sustainability motivations, future work needs to investigate rancher land management practices (i.e., grazing management plans) and determine how these management strategies relate to the willingness to join future sustainability practices.

## Supplementary Material

txac094_suppl_Supplementary_Material
