# Supplementary material for: Beef Quality Assurance national rancher survey: program participation, best management practices, and motivations for joining future sustainability programs
Source: Transl Anim Sci. 2022 Aug 1;6(3):txac094. doi: 10.1093/tas/txac094 (PMC9341740; doi:10.1093/tas/txac094)
Supplement: txac094_suppl_Supplementary_Material [file txac094_suppl_supplementary_material.docx]

Ranch Management Practices-Questions

Q: What is your current age?

- 18-30 (1)
- 30-40 (2)
- 40-50 (3)
- 50-60 (4)
- 60-70 (5)
- 70 and older (6)

Q: How many years have you personally been ranching? (Since you were 18 years old)

- 0-4.9 Years (1)
- 5-9.9 years (2)
- 10-19.9 years (3)
- 20-29.9 years (4)
- More than 30 years (5)

Q: What is your primary zip code?

________________________________________________________________

Q: What is your gender?

- Male (1)
- Female (2)

Q: On average (over the last 5 to 10 years), how many animals of each livestock type graze on land you own or personally lease?

|  | Total Number of Animals (1) |
| --- | --- |
| Number of cows and yearling heifers (1) |  |
| Number of stockers (2) |  |
| Number of bulls (3) |  |
| Number of sheep (4) |  |

Q: Please describe your grazing land.

|  | Acres of Land (1) |
| --- | --- |
| Total acres personally owned for grazing (1) |  |
| Total privately owned acres leased for grazing (2) |  |
| Total public acres leased for grazing, public (ex. Forest Service land) (3) |  |
| Total irrigated acres for grazing (owned or leased) (4) |  |
| Total acres managed for grazing (owned, leased, and irrigated) (5) |  |

Q Are you a first or multi-generation rancher?

- First generation rancher (1)
- Multi-generation rancher (2)

Q: Do you have a succession plan for your ranch that identifies a strategy for keeping land in ranching in the future?  (ex. pass on to family for ranching)

- Yes (1)
- No (2)
- In progress (3)
- Not applicable (Do not own land) (4)

Q: What is your percent of your total income coming from the ranch (beef cattle only)?

- 1-25% (1)
- 26-50% (2)
- 51-75% (3)
- 76%-100% (4)

Q: Does your operation include other activities that affect land management? (Check all that apply)

- Other agricultural production (1)
- Passive recreation (ex. hiking, birding, horseback riding) (2)
- Conventional energy development (ex. oil, coal, natural gas) (3)
- Extractive Recreation (ex. hunting, fishing) (4)
- Alternative energy development (ex. solar, wind, biofuel) (5)
- Special Events (ex. weddings, parties) (6)
- Other (7)

Q: Do you participate in any ranching certification programs, if so which ones? (Check all that apply)

|  | Program (1) |
| --- | --- |
| I do not participate in a ranching certificate program (1) |  |
| Humanely Raised (2) |  |
| 100% Grass-Fed or Grass-Finished (3) |  |
| All Natural (4) |  |
| Beef Quality Assurance (BQA) (5) |  |
| Certified Organic (6) |  |
| Verified Source and Age (7) |  |
| Non-hormone Treated Cattle (NHTC) (8) |  |
| Global Animal Partnership (GAP) (9) |  |
| Other (10) |  |

Q: Do you participate in any government landowner assistance program? [ex. USDA or Natural Resource Conservation Service (NRCS), such as the Environmental Quality Incentives Program (EQIP), Conservation Stewardship Program (CSP)]

- Yes (If yes, please fill in program below) (1) ________________________________________________
- No (2)

Q: Which of the following best describes your participation in the Beef Quality Assurance (BQA) program?

- I have not participated in BQA (5)
- I went to a BQA program, but I never became BQA certified (4)
- I was BQA certified at one time, but never re-certified (3)
- I am currently certified (I first enrolled in BQA within the last 3 years) (1)
- I am currently certified (I have been in BQA for over 3 years and I have re-certified at least once) (2)

|  |
| --- |

Q: If you participated in Beef Quality Assurance (BQA), what were your top 3 reasons for joining the program? (Please select up to **3 choices** from the following options.)

- BQA animals fetch a higher price (1)
- Neighbors/competitors were also performing BQA practices (2)
- Improve animals’ health and welfare (3)
- Increases longevity of your operation (4)
- Believed voluntary participation would prevent regulatory requirements (5)
- Reputation of my operation is greater when animals are a part of BQA (6)
- Consumer perceptions/demand concerns about animal welfare (7)

Q: If you have participated in BQA, do you believe that adopting BQA practices was beneficial to your ranching operation?

- Extremely beneficial (1)
- Very beneficial (2)
- Beneficial (3)
- Somewhat beneficial (4)
- Not beneficial (5)

Q:  If you have participated in BQA, are there any sections in the BQA guidelines that you did not find feasible or helpful for your operation? Please write your answer below and explain why the section(s) were not feasible and/or helpful. (Sections include, antibiotic/vaccination procedures, weaning practices, and transportation procedures).  Please write your answer below.

________________________________________________________________

Q: If you have **NOT**participated in Beef Quality Assurance (BQA),  please select from the following statements below as to why you did not join BQA. (Check all that apply)

- I was not aware of the BQA program (1)
- No BQA certification opportunities in my area (2)
- Time commitment too high (3)
- Not enough financial reward (4)
- BQA practices do not make sense or are confusing (5)
- My operation exceeds BQA standards and so I do not believe it is necessary (6)
- Practices do not fit my operation goals or management strategies (7)
- Other (If other, please explain) (8) ________________________________________________

Q:Please select your 3 top reasons why you would adopt a new ranching practice. (Check up to 3 boxes that apply)

- It is profitable (1)
- It improves animal health (3)
- Would limit government involvement (5)
- Expected benefits greatly out weigh cost and effort to implement (2)
- It improves environmental health (4)
- It improves my quality of life (6)
- Another rancher/ranching friend has recommended the program (7)
- It is recommended by national/state/local cattlemen’s organization(s) (8)

Q: Please select your **3 top reasons** why you would **NOT** adopt a new ranching practice. (Check up to 3 boxes that apply)

- It would require more time investment (1)
- Does not fit your production system (2)
- Requires certification (3)
- Requires re-certification (4)
- Requires audits (5)
- Expected cost outweighs expected benefits (6)
- Benefits are short term (5 years or less) (7)
- Does not align with your values (8)
- Other (If other, please explain) (9) ________________________________________________

Q: Where do you give vaccine/ hormone/ antibiotic injections for beef cattle? You may choose more than one location for each injection type.

|  | Shoulder (1) | Rump (2) | Tailhead (3) | Neck (4) | Other (5) |
| --- | --- | --- | --- | --- | --- |
| Antibiotics (1) |  |  |  |  |  |
| Vaccines (2) |  |  |  |  |  |
| Reproductive hormones (ex. estroplan, P.G. 600) (3) |  |  |  |  |  |

Q: When managing your herd, what procedures do you use/perform? (Check all that apply)

- Fence line wean, utilizing a fence to separate the cow from the calf (1)
- Waiting 45 days after weaning to ship calves (2)
- Ship calves immediately or very shortly after weaning (3)
- Ship calves 7 to 45 days after weaning (4)
- Castrate before 3 months of age (5)
- Castrate after 3 months of age (6)
- Darting, using remote devices to give injections (7)
- Established a herd health development program with your vet (8)
- Other methods (If other, please explain) (9) ________________________________________________

Q: Would participating in a non-mandatory "beef sustainability" rancher education program be beneficial for your operation?

- Yes (1)
- No (2)
- Unsure (3)
